# The initial and lingering impact of coronavirus disease 2019 (COVID-19) on catheter-associated infections in a large healthcare system in New York City

**DOI:** 10.1017/ash.2022.223

**Published:** 2022-05-04

**Authors:** Jevon Samaroo-Campbell, Wenqi Qiu, Habtamu Asrat, Marie Abdallah, Mary Fornek, Briana Episcopia, John Quale

**Affiliations:** 1 Division of Infectious Diseases, NYC Health and Hospitals Kings County, Brooklyn, New York; 2 School of Public Health, SUNY Downstate Medical Center, Brooklyn, New York; 3 Department of Infection Control and Prevention, NYC Health and Hospitals Central Office, New York, New York; 4 Department of Infection Control and Prevention, NYC Health and Hospitals Kings County, Brooklyn, New York

## Abstract

Catheter-related infections increased during surges of coronavirus disease 2019 (COVID-19) in an 11-hospital system in New York City. A disproportionate number of central-line infections occurred in larger hospitals. Patients with COVID-19 had shorter times from catheter insertion to infection and a higher incidence of infections with enterococci.

Prior to the coronavirus disease 2019 (COVID-19) pandemic, considerable progress was reported in reducing catheter-related infections.^1–3^ With the initial surge of COVID-19, national data from the National Healthcare Safety Network (NHSN) documented a 28% increase in standardized infection ratios (SIRs) for central-line–associated bloodstream infections (CLABSIs) in the second quarter of 2020.^4^ More recently, the NHSN reported greater SIRs for both CLABSIs and catheter-associated urinary tract infections (CAUTIs) during July–December 2020.^5^

In this report, we detail the impact of COVID-19 on reportable catheter-associated infections in a public hospital system in New York City. Prior to the pandemic, a disproportionate number of infections was previously noted in the larger centers.^1^ We also examined the effect of the pandemic on this disparity.

## Methods

The NYC Health and Hospitals Enterprise includes 11 public acute-care hospitals. The 5 larger hospitals each had >8,000 admissions from January through June 2021. We retrospectively reviewed information from the NHSN database regarding hospital-specific reportable infections. Line listings for patients with healthcare-associated CLABSIs and CAUTIs were obtained.

The Student *t* test and the Fisher exact test were used to compare continuous and categorical values, respectively. This study was approved by the SUNY Downstate Medical Center Institutional Review Board and the H+H System to Track and Approve Research program.

## Results

For the 11-hospital system, CLABSI infection rates correlated with the burden of the pandemic. At baseline in 2019, there were 1.07 CLABSIs per 1,000 catheter days. During January–July 2020, the infection rate rose to 3.21 infections per 1,000 catheter days, when 27.6% of the inpatient census comprised patients with COVID-19. During July–December 2020, there were 1.69 infections per 1,000 catheter days, and patients with COVID-19 comprised 3.54% of the census. During January–June 2021, when 11.26% of the inpatient census were COVID-19 patients, there were 1.85 infections per 1,000 catheter days. In contrast, we observed a steady increase in the CAUTI infection rates. During 2019, there were 1.20 CAUTIs per 1,000 catheter days. For the sequential 6-month intervals from January 2020 through June 2021, the infection rates were 1.78, 2.00, and 2.15 CAUTIs per 1,000 catheter days.

### CLABSIs

From January 1, 2019, to June 30, 2021, there were 410 CLABSIs in 388 patients. In total, 228 CLABSIs (56%) occurred in patients in intensive care units. Of these 388 patients, 173 were admitted for COVID-19. Compared to patients without COVID-19, patients with COVID-19 were more likely to be male (68% vs 58%; *P* = .05), were more likely to be Hispanic (44% vs 27%; *P* < .001) or of Asian descent (27% vs. 8%, *P* < .001), and were less likely to be Black (55% vs 74%; *P* < .001). Mortality rates were higher in patients with COVID-19 (60% vs 38%; *P* < .0001).

For the 15 months prior to the onset of the pandemic, the quarterly catheter infection rates and SIRs averaged 1.09±0.43 infections per 1,000 catheter days and 0.98±0.38, respectively. During the subsequent 15 months, infection rates and SIRs rose to 1.76±0.93 infections per 1,000 catheter days and 1.47±0.76, respectively. An increased number of blood cultures drawn during the pandemic did not account for this difference; at 5 hospitals, 20,166 blood cultures were drawn from July–December 2019, and 22,224 cultures were drawn from January through June 2020.

Furthermore,423 central catheters were involved in the 410 infections (Table 1). More CLABSIs involved short-term catheters in patients with COVID-19, and more CLABSIs involved dialysis and peripherally inserted catheters in patients without COVID-19.

**Table 1.** Types and Locations of Catheters Associated With CLABSIs January 2019–June 2021 Among 11 Acute-Care Medical Centers

Variable	Total No.	Catheters in COVID-19 Patients (n=185)	Catheters in Patients Without COVID-19 (n=238)	*P* Value
		No. (%)	No. (%)	
**Type of catheter**				
Short term	247	153 (83)	94 (39)	<.001
Dialysis	98	27 (14)	71 (30)	.003
Peripherally inserted	69	5 (3)	64 (27)	<.001
Chemo port	4	0 (0)	4 (2)	.14
Umbilical	5	0 (0)	5 (2)	.07
**Location of catheter**				
Brachial	62	5 (3)	57 (24)	<.001
Femoral	69	36 (19)	33 (14)	.14
Jugular	220	109 (59)	111 (47)	.01
Subclavian	67	35 (19)	32 (13)	.41
Umbilical	5	0 (0)	5 (2)	.07

Time from admission to infection for patients with COVID-19 was 20.5±22.8 versus 42.6±82.7 days for patients without COVID-19 (*P* = .0001). Time from device insertion to infection was 9.9±6.0 for patients with COVID-19versus 17.6±23.2 days for patients without COVID-19 (*P* < .001). Hospital length of stay was shorter in patients with COVID-19 (47.5±48.7 for patients with COVID-19 vs 80.5±115.6 days for patients without COVID-19; *P* = .001).

Also, 443 pathogens were involved in these 410 infections. In patients with COVID-19, *Enterococcus faecalis* (30% vs 12%; *P* < .001) and *E. faecium* (15% vs 10%; *P* = .08) occurred more frequently and *Escherichia coli* (2% vs 8%; *P* = .002) and *Pseudomonas aeruginosa* (0.5% vs 4%; *P* = .02) occurred less frequently.

### CAUTIs

From January 1, 2019, to June 30, 2021, there were 364 CAUTIs in 347 patients. In total, 172 CAUTIs (47%) occurred in patients located in intensive care units. Of the 347 patients, 93 were admitted with COVID-19. Compared to patients without COVID-19, those with COVID-19 were more likely to be Hispanic (18% vs 9%) or Asian (29% vs 5%). In-hospital mortality rates were significantly higher in the patients with COVID-19 (39%) than in patients without COVID-19 (22%; *P* = .004). Time from admission to infection for patients with COVID-19 was 24.9±17.0 versus 29.9±39.9 days for patients without COVID-19 (*P* = .095), and time from device insertion to infection was 14.9±12.3 for patients with COVID-19 versus 23.2±36.9 days for patients without COVID-19 (*P* = .001). Patients with COVID-19 had average lengths of hospital stay (30.8±27.6 days) similar to those of patients without COVID-19 (38.6±62.3 days).

Compared to the 15 months prior to the onset of the pandemic, the infection rates during the pandemic increased from 1.03±0.18 to 1.80±0.21 infections per 1,000 catheter days (*P* = .0003) and SIRs increased from 0.78±0.16 to 1.33±0.17 (*P* = .0007).

Overall, 382 pathogens were involved in the 364 infections. In patients with COVID-19, infections were more frequently *E. faecalis* (22% vs 7%; *P* = .001) and *E. faecium* (18% vs 7%; *P* = .003) and were less commonly *P. aeruginosa* (8% vs 17%; *P* = .03).

### Larger versus smaller hospitals

From March 26 through April 18, 2020, there were 684 transfers (28.5 per day) within the hospital system; 414 (61%) of these patients were received at large hospitals. From January 1 through April 30, 2021, there were 833 interhospital transfers (6.9 per day). Of these transfers, 243 patients (29%) were received at large hospitals.

In 2020, the number of CLABSIs increased disproportionately in the larger hospitals (Table 2). During 2020, 55 CLABSIs occurred in patients who had been transferred previously: 49 patients (30 patients with COVID-19) had been transferred to large hospitals, and 6 patients (4 patients with COVID-19) had been transferred to small hospitals. By the first half of 2021, the discrepancies between the larger and smaller hospitals vanished, with a marked increase of CLABSIs observed in the smaller hospitals. During the first 6 months of 2021, 27 CLABSIs occurred in patients who had been transferred previously: 26 patients had been transferred to large hospitals (15 patients with COVID-19) and 1 had been transferred to a small hospital.

**Table 2.** Trends in Central-Line–Associated Bloodstream Infections (CLABSIs) and Catheter-Associated Urinary Tract Infections (CAUTIs) in Large and small Hospitals from January 2019 through June 2021

Variable	Hospital Size	Jan–Jun 2019	Jul–Dec 2019	Jan–Jun 2020	Jul–Dec 2020	Jan–Jun 2021
**CLABSIs**						
No. of CLABSIs in COVID-19 patients	Large	31	33	155 (119)	54 (5)	65 (26)
	Small	9	9	23 (11)	7 (0)	24 (16)
Rate (per 1000 catheter days)	Large	1.45±0.45	1.27±0.63	4.57±1.23	2.15±1.08	1.86±1.36
	Small	0.61±0.55^a^	0.42±0.41^a^	1.14±1.39^a^	0.47±0.46^a^	1.26±0.7
Rate (per 10000 patient days)	Large	1.25±0.24	1.31±0.76	7.67±2.79	2.41±1.75	2.48±2.24
	Small	0.61±0.53^a^	0.44±0.44	1.64±1.78^a^	0.53±0.51	1.46±1.38
Standardized infection ratio	Large	1.32±0.40	1.13±0.51	3.36±1.11	1.84±0.88	1.41±0.91
	Small	0.61±0.55	0.42±0.41^a^	1.21±1.58^a^	0.47±0.44^a^	0.97±0.71
**CAUTIs**						
No. of CAUTIs in COVID-19 patients	Large	25	31	69 (43)	56 (5)	69 (24)
	Small	14	17	30 (11)	19 (1)	34 (14)
Rate (per 1,000 catheter days)	Large	1.13±0.43	1.34±1.33	2.08±0.77	2.26±1.59	2.21±1.36
	Small	0.98±0.50	0.73±0.79	1.11±1.15	1.39±0.60	1.75±0.99
Rate (per 10,000 patient days)	Large	0.97±0.36	1.89±1.19	3.69±2.04	2.44±1.73	2.67±1.70
	Small	0.90±0.55	0.80±0.95	1.88±2.02	1.45±0.82	2.27±1.54
Standardized infection ratio	Large	0.81±0.32	0.94±0.90	1.28±0.36	1.50±0.96	1.53±0.85
	Small	0.86±0.53	0.60±0.63	0.73±0.65	1.18±0.56	1.33±0.65

a *P* < .05 compared to the large hospitals.

Regarding CAUTIs, both large and small hospitals were adversely affected by the pandemic (Table 2). During 2020, 14 CAUTIs occurred in patients who had been transferred previously, of whom 12 patients (including 3 patients with COVID-19) were received at large hospitals. During the first 5 months of 2021, 26 CAUTIs occurred in patients who had been transferred previously; all were received at the large hospitals and 11 of these patients had been admitted for COVID-19.

## Discussion

In this report, the initial surge in New York City during the first 6 months of 2020 was associated with a 345% increase in the number of CLABSIs when compared to the same period in 2019. Increases were also documented for the periods July–December 2020 and January–June 2021. Increased infections have been attributed to reduced skin-decolonization procedures, interruptions in dressing or line integrity related to prone positioning of patients, and frequent blood draws through central lines.^4,6–8^ The use of extension tubing to allow for adjustment of pumps outside patient rooms may have also contributed to a decrease in catheter checks.^9^ Both staffing and personal protection equipment shortages have been proposed as contributory factors for increased catheter-associated infections.^4,7,8,10^ From February 2020 to May 2021, decreases in registered and licensed practical nurses occurred in the NYC Health + Hospitals Enterprise (www.osc.state.ny.us/files/reports/osdc/pdf/report-9-2022.pdf). Finally, the responsibilities of infection preventionists changed considerably during COVID-19 surges. More time was dedicated to monitoring isolation of patients, creation of dedicated COVID-19 units, training and distribution of protective equipment, and reporting COVID-19–related data, and less time was dedicated to routine activities (including monitoring and enforcing bundles regarding catheter care).

Recent data from the NHSN indicated that, compared to infections in the pre–COVID-19 period, the time to infection increased during COVID-19 surges.^5^ In our study, however, the time from catheter insertion to time of central-line infection was significantly shorter in patients with COVID-19. Short time intervals to infection may be related to deviations in sterile technique during insertion, especially if central lines are placed under emergent conditions.^7^ As noted above, frequent blood draws through the central line as well as dressing and line compromise during the prone position likely contributed to the shorter time to infection.

Compared to CLABSIs, data from the NHSN documented more modest increases in the rates of CAUTIs attributed to the pandemic, with a progressive rise throughout 2020.^5,6^ In this report, a 154% increase in the number of CAUTIs occurred during the first 6 months of 2020. Through the first 6 months of 2021, the number of CAUTIs has not returned to baseline, and many CAUTIs have occurred in patients without COVID-19. As with CLABSIs, prone positioning and reduced patient contact have been reported to be contributing factors.^6^ The increased number of infections in patients without COVID-19 has been attributed to the greater demands placed on the healthcare system by the pandemic.^7^ The shorter time from catheter placement to infection in patients with COVID-19 may again be secondary to urinary catheter placement under emergent situations.

Given the marked increases in catheter-associated infections during surges of COVID-19, the call to maintain infection prevention processes regarding device use is well heeded.^8^
